# Successful azithromycin desensitization using a modified protocol after initial reaction: A pediatric case report

**DOI:** 10.1016/j.idcr.2026.e02635

**Published:** 2026-06-13

**Authors:** Ahmad Almtrafi, Bashayer Habib, Rayan Al Lohaibi, Nouf Alsahaf, Sara Abed, Loie Goronfolah, Lein Azzhary, Mona Aldabbagh, Ahmad M. Alghamdi, Samiyah Althagafi, Mohammed A. Almatrafi

**Affiliations:** a Division of Pediatric Infectious Disease, Department of Pediatrics, King Abdullah Specialized Children’s Hospital, MNGHA, King Abdullah International Medical Research Center, Jeddah, Saudi Arabia; b Division of Pediatric Allergy Immunology, Department of Pediatrics, King Abdullah Specialized Children’s Hospital, MNGHA, King Abdullah International Medical Research Center, Jeddah, Saudi Arabia; c Department of Pediatrics, King Abdullah Specialized Children’s Hospital, MNGHA, King Abdullah International Medical Research Center, College of Medicine, King Saud bin Abdulaziz University for Health Sciences, Jeddah, Saudi Arabia; d Division of Pediatric Infectious Disease, Department of Pediatrics, King Abdullah Specialized Children’s Hospital, MNGHA, King Abdullah International Medical Research Center, College of Medicine, King Saud bin Abdulaziz University for Health Sciences, Jeddah, Saudi Arabia; e Department of Infection prevention and control, King Saud Bin Abdulaziz University for Health Sciences, King Abdulaziz Medical city-WR, Jeddah, Saudi Arabia; f Department of Pediatrics, College of Medicine, Umm Al-Qura University, Makkah, Saudi Arabia

**Keywords:** Bordetella infection, Whooping cough, Macrolides, Azithromycin, Anaphylactic reaction

## Abstract

Pertussis is a highly contagious infection associated with significant morbidity and mortality in young infants despite widespread vaccination programs. Early recognition and treatment are important to reduce disease severity, shorten symptom duration, and limit transmission. We report the case of a 51-day-old previously healthy boy who presented with a 4-day history of whooping cough, apnea, circumoral cyanosis, post-tussive emesis, and reduced oral intake. Pertussis was confirmed by a nasopharyngeal respiratory multiplex PCR panel detecting *Bordetella pertussis*. The patient initially received two doses of oral azithromycin (10 mg/kg/dose); however, approximately 20–30 min after the second dose, he developed facial puffiness and lip swelling consistent with a probable immediate hypersensitivity reaction. This complicated management because alternative antimicrobial agents, including trimethoprim-sulfamethoxazole and fluoroquinolones, were considered unsuitable owing to age-related safety concerns and limited clinical applicability. Following multidisciplinary evaluation and individualized risk–benefit assessment, azithromycin desensitization was undertaken. The initial desensitization attempt was discontinued because of recurrent hypersensitivity symptoms, prompting modification of the protocol. A second desensitization attempt using extended infusion and observation intervals was successfully completed. Following tolerance of intravenous azithromycin, the patient was transitioned back to oral therapy and completed the full 5-day treatment course without recurrence of hypersensitivity symptoms or delayed reactions. His cough gradually improved, and apnea episodes resolved before discharge. This case highlights azithromycin desensitization as a potential individualized therapeutic strategy in carefully selected infants with pertussis and suspected macrolide hypersensitivity when safer alternative treatment options are limited. Further evidence is required before broader conclusions regarding safety, efficacy, and reproducibility can be drawn.

## Introduction

Pertussis, commonly known as whooping cough, is a bacterial infection caused by *Bordetella pertussis* [1]. The clinical presentation varies depending on age and immunity, with the classical presentation typically observed in children and unimmunized individuals [2]. Higher mortality and morbidity have been seen in younger infants diagnosed with pertussis [3], [4]. Early identification and prompt treatment initiation are crucial, particularly in light of increasing regional reports of infants diagnosed with malignant pertussis, a severe form of disease typically characterized by marked leukocytosis, pulmonary hypertension, respiratory failure, and high mortality [5], [6].

Antibiotic therapy with macrolide agents is recommended for all patients with a clinical or microbiologic diagnosis of pertussis, as it shortens symptom duration, reduces transmission to susceptible contacts, and may lessen disease severity when initiated early in the course of illness [7], [8]. Among macrolides, azithromycin is generally preferred in infants younger than 2 months because of its favorable safety profile, shorter treatment duration, and lower reported association with infantile hypertrophic pyloric stenosis compared with erythromycin, while clarithromycin is not routinely recommended in this age group due to limited safety data [2].

Although it is uncommon in children, macrolide hypersensitivity may lead clinicians to choose alternative therapy if not contraindicated [9]. Trimethoprim-sulfamethoxazole is generally avoided in infants younger than 2 months because of safety concerns, limiting alternative antimicrobial options in this age group [7]. This case highlights the successful management of a 51-day-old boy who underwent successful azithromycin desensitization for the treatment of pertussis after developing signs and symptoms concerning for anaphylactic reaction following the initial use of azithromycin.

## Case presentation

A 51-day-old previously healthy boy presented to the emergency department with a 4-day history of whooping cough associated with brief apnea episodes lasting a few seconds, circumoral cyanosis, post-tussive emesis, reduced oral intake, and decreased activity. He did not require supplemental oxygen, with oxygen saturation remaining > 94% on room air at presentation. Birth history was unremarkable. His immunization is up to date based on Saudi vaccination schedule, which includes receiving the 1st dose of Hepatitis B vaccine at birth. He lives with his parents and three siblings. Exposure history notable for sick family members with upper respiratory tract infections. His mother did not receive Tetanus, Diphtheria and Pertussis vaccine during her pregnancy. The family had no pets at home. No recent travel. His diet consisted of cow milk-based formula, and he was achieving his age-appropriate milestone.

At presentation, vital signs were within normal limits for age; temperature (37.1 C), Respiratory rate (41/min), heart rate (166/min), and oxygen saturation (>94% room air). He was well perfused, sleeping comfortably with subcostal retraction. The physical examination was otherwise unremarkable. Laboratory testing revealed leukocytosis (22.6 ×10^9/l) with absolute lymphocytosis (17.310^9/l). Blood cultures were reported negative.

He was admitted with suspected pertussis after nasopharyngeal respiratory multiplex PCR testing detected *Bordetella pertussis.* He received two doses of oral azithromycin (47 mg; 10 mg/kg/dose). However, After the second dose of azithromycin, the patient developed acute facial puffiness and lip swelling ** min of administration**, consistent with an immediate-type hypersensitivity reaction. No urticaria, stridor, wheezing, vomiting, or hypotension were observed, and oxygen saturation remained > 94% on room air without respiratory compromise. Although adrenaline was not required, the reaction was clinically significant given the rapid onset, mucosal involvement, and progression of swelling. These findings met the **World Allergy Organization (WAO) criteria for a probable IgE-mediated immediate hypersensitivity reaction**, prompting discontinuation of azithromycin and consultation with the allergy/immunology service for desensitization. Given the significant history of allergic reaction to azithromycin and no other alternative options available for pertussis in this age group, allergy and immunology service consultation was made for macrolide desensitization. Prior to desensitization, the allergy/immunology team performed a structured risk assessment. Formal skin testing was **not pursued**, as validated skin-testing reagents for azithromycin are not standardized and have limited diagnostic reliability in infants. A multidisciplinary discussion involving pediatric infectious diseases, allergy/immunology, and critical care teams reviewed the risks and benefits of desensitization versus withholding therapy. The infant’s parents received **detailed counseling**, including explanation of potential risks (including recurrent hypersensitivity, anaphylaxis, and need for escalation of care), expected monitoring procedures, and alternative management options. **Written informed consent** was obtained before proceeding. Desensitization was performed in a pediatric critical care setting with continuous cardiorespiratory monitoring. The patient had **two secured peripheral IV lines**, one dedicated to azithromycin infusion and the other reserved for emergency medications. **Rescue therapies**, including intramuscular adrenaline, IV antihistamines, corticosteroids, and fluid boluses, were immediately available at the bedside. A pediatric intensivist and allergy specialist were present during the initial steps. Discontinuation criteria included development of respiratory compromise, hypotension, generalized urticaria, persistent vomiting, or progression of mucosal swelling. The protocol followed a modified 12-step incremental dosing schedule based on Castells et al., with adjustments to infusion duration and rest intervals after the initial reaction during step 5. The protocol was generated based on Castells and colleagues’ protocol; a standardized 12-step protocol using three IV solutions with different target drug concentrations [10].

The target azithromycin dose of 47 mg was calculated based on the patient’s body weight of [4.7] kg and the recommended azithromycin dosing regimen of [10 mg/kg/day] for pertussis in infants. Three IV solutions of 125 ml normal saline were used and care was provided in critical care setting. The first solution was injected with 1% of the targeted Azithromycin dose (47 mg), the second IV solution was injected with 10% of the targeted dose and the third IV solution was injected with the remaining dose (Table 1). Each IV solution was given in four different steps. The rate of infusion was increased every 30 min to administer double the dose of the previous step (Table 2). Step 12 was intentionally longer than preceding steps because it delivered the remaining bulk of the target dose. The duration was calculated to ensure complete administration of the final fraction of the total 47 mg dose while maintaining a controlled infusion rate appropriate for the patient’s weight and clinical status.

**Table 1:** Azithromycin concentration used for desensitization protocol.

Full dose	47 mg	mg/ml	Total mg to be injected each solution
Solution 1	125 ml	0.00376	0.47
Solution 2	125 ml	0.0376	4.7
Solution 3	125 ml	0.376	47

**Table 2:** A 12-step azithromycin desensitization protocol for a target dose of 47 mg with an infusion rate over 30 min in each step.

STEP	Solution	Rate (ml/hr)	Time(min)	dose(mg)	Dose (mg/kg)	Cumulative dose (mg)
1	1	1	30	0.0018	0.00038 mg/kg	0.0018
2	1	2.5	30	0.0045	0.00096 mg/kg	0.0063
3	1	5	30	0.009	0.00191 mg/kg	0.0153
4	1	10	30	0.018	0.00383 mg/kg	0.033
5	2	2.5	30	0.045	0.00957 mg/kg	0.078
6	2	5	30	0.09	0.01915 mg/kg	0.168
7	2	10	30	0.18	0.03830 mg/kg	0.348
8	2	20	30	0.36	0.07660 mg/kg	0.708
9	3	5	30	0.9	0.19149 mg/kg	1.6
10	3	10	30	1.8	0.38298 mg/kg	3.4
11	3	20	30	3.6	0.76596 mg/kg	7
12	3	40	166	40	8.51064 mg/kg	47

All azithromycin solutions were prepared from the standard commercially available intravenous formulation. Each solution was reconstituted and diluted according to manufacturer specifications using sterile normal saline under pharmacy supervision. No compounded or off-label preparations were used. This standardized preparation process is essential for ensuring reproducibility, stability, and safety during desensitization.

**Fig. 1:**
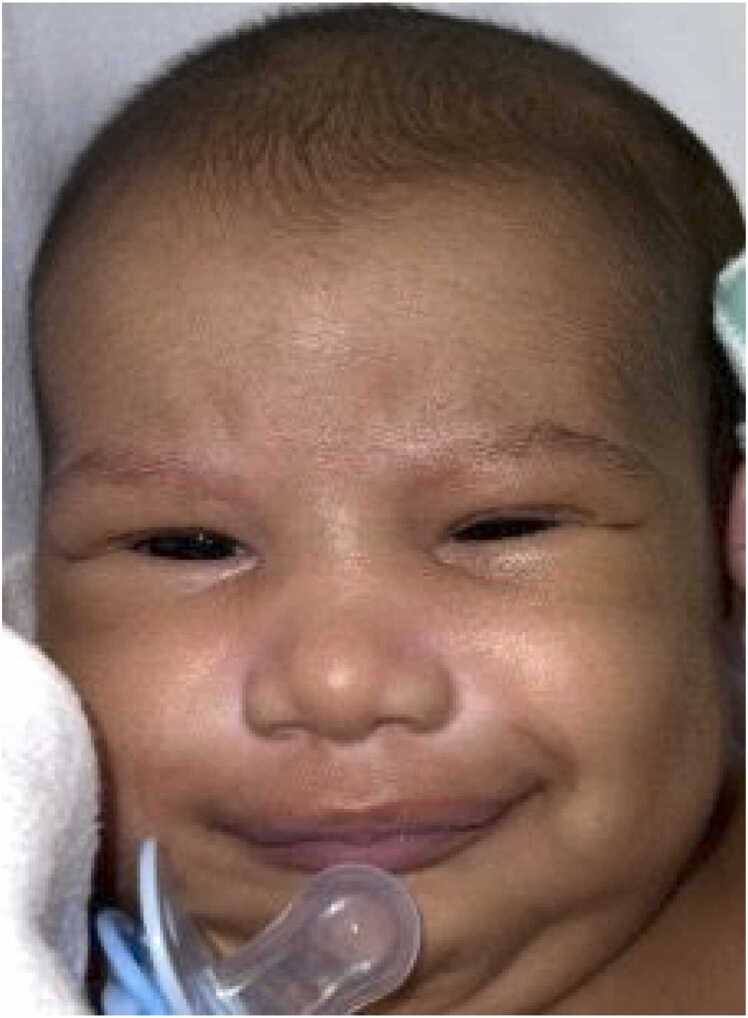
Facial erythema and puffiness along with lip swelling developed during step 5 of azithromycin desensitization protocol.

The process was aborted given the development of another allergic reaction during step 5 **(**Fig. 1**)** even with starting him on diphenhydramine and methylprednisolone. The reaction occurred during step 5 of the first desensitization attempt, approximately 20–30 min after infusion initiation. After treatment with diphenhydramine and methylprednisolone and a 2–3-hour period of clinical stability, the protocol was restarted the next day using the modified extended-interval schedule (Table 3).

**Table 3:** A 12-step azithromycin desensitization protocol for a target dose of 47 mg with an infusion rate over 45 min with rest intervals.

Step	Solution	Rate (ml/hr)	Time(min)	dose(mg)	Dose (mg/kg)	Cumulative dose (mg)
1	1	1	45	0.0018	0.00038 mg/kg	0.0018
2	1	2.5	45	0.0045	0.00096 mg/kg	0.0063
3	1	3.5	45	0.0098	0.00209 mg/kg	0.0161
4	1	7	45	0.0197	0.00419 mg/kg	0.0358
5	2	1.5	45	0.042	0.00894 mg/kg	0.078
6	2	3.5	45	0.098	0.02085 mg/kg	0.175
7	2	7	45	0.197	0.04191 mg/kg	0.372
8	2	14	45	0.39	0.08298 mg/kg	0.762
9	3	3.5	45	0.98	0.20851 mg/kg	1.742
10	3	7	45	1.97	0.41915 mg/kg	3.71
11	3	14	45	3.94	0.83830 mg/kg	7.65
12	3	28	225	39.5	8.40425 mg/kg	47

Doses listed in Table 2, Table 3 represent the calculated amount of ***azithromycin base*** delivered per step, derived from the infusion rate, duration, and solution concentration. Rate–time–dose calculations were cross-checked for internal consistency

**Fig. 2:**
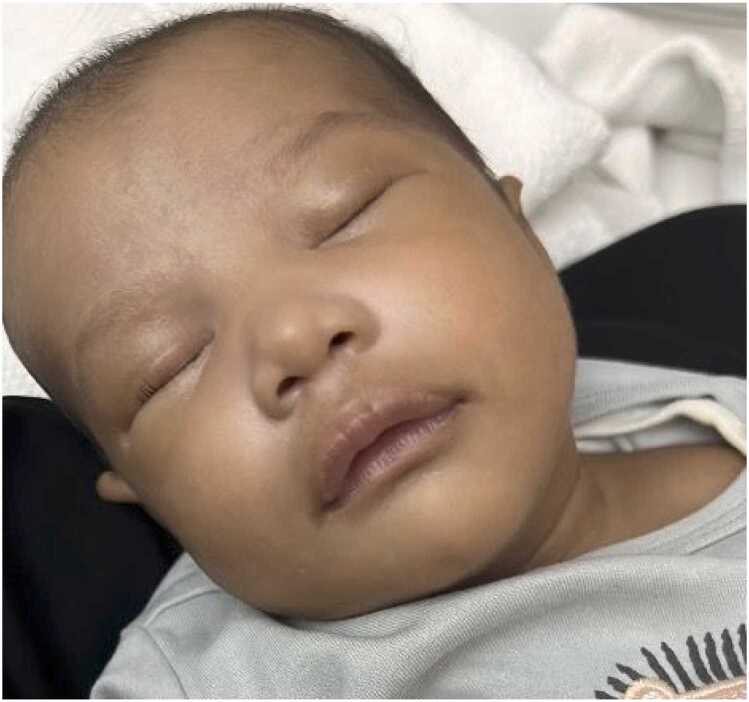
Patient tolerated the azithromycin course after desensitization protocol with no evidence of allergic reaction.

The patient was kept on diphenhydramine and methylprednisolone and desensitization protocol was repeated. However, this time, the infusion duration in each step was increased from 30 min to 45 min with 45 min rest intervals between each step (Table 3). He completed the desensitization process successfully. His allergic reaction manifestation including periorbital swelling, eye puffiness and lip swelling subsided on the second day of desensitization. He was transferred back to the general pediatric ward on day 3 of hospitalization. Following successful completion of the desensitization protocol and tolerance of the intravenous doses, the final 2 days of therapy (2 doses) were administered orally **(**Fig. 2**).**

The patient completed the full 5-day azithromycin course without recurrence of swelling, rash, respiratory symptoms, or delayed hypersensitivity reactions. No late-phase reactions were observed during hospitalization or in the 72 h following the final dose. His cough gradually improved, and apnea episodes resolved prior to discharge.

## Discussion

Pertussis is a highly contagious bacterial infection mainly caused by *Bordetella pertussis* strain [1]. Infants under 6 months are particularly vulnerable to complications and mortality from the condition [11]. About 50% of infants diagnosed with pertussis require hospitalization within their first year of life, with a mortality rate of 1–2% [12]. Early recognition and treatment of pertussis is crucial; however, it might be challenging given the possibility of atypical presentation in infants under 6 months of age [8]. Compared to older children, upper respiratory symptoms, with or without the presence of apnea, may constitute the sole presenting symptoms in this age group [7].

The management of whooping cough consists of supportive care and macrolide antibiotic agents. Azithromycin is the preferred antibiotic agent over erythromycin and clarithromycin for infants under two months of age due to its lower side effect profile and shorter duration of therapy. The latter two agents are associated with an increased risk of infantile hypertrophic pyloric stenosis and should be used with caution in this age group. TMP-SMX is a plausible alternative agent for treating pertussis; however, it is contraindicated in infants under 2 months due to an increased risk of kernicterus [7].In our patient, given his young age, apnea, circumoral cyanosis, and reduced oral intake, withholding antimicrobial therapy was considered less favorable because of the potential risk of disease progression.

The development of a reaction concerning for anaphylaxis following azithromycin exposure created a separate therapeutic challenge related to drug hypersensitivity management. In this context, the clinical question shifted from standard pertussis treatment to whether azithromycin could be safely reintroduced despite suspected hypersensitivity. Drug desensitization is a specialized strategy that allows temporary induction of tolerance through administration of gradually increasing drug doses under close monitoring [13], [14]. Given the absence of safer age-appropriate antimicrobial alternatives, a multidisciplinary discussion involving pediatric infectious diseases, allergy/immunology, and critical care teams supported proceeding with carefully monitored azithromycin desensitization.

Published evidence regarding macrolide desensitization in pediatric patients, particularly infants and young children, remains extremely limited and is primarily confined to isolated case reports, which substantially limits conclusions regarding reproducibility, safety, and efficacy. To our knowledge, no previously identified reports have specifically described intravenous azithromycin desensitization in an infant with pertussis. Existing literature consists mainly of isolated adult and pediatric case reports involving macrolide hypersensitivity in other clinical contexts [9], [13], [15]. Swamy et al. described successful clarithromycin and azithromycin desensitization in an adult patient [13], while Holmes et al. reported similar outcomes [15]. Petitto et al. described successful clarithromycin desensitization in an 11-year-old child with osteomyelitis secondary to nontuberculous mycobacterial infection [9].

However, extrapolation of findings from adult patients and older children to a 51-day-old infant requires substantial physiological and clinical caution, given the marked differences in immune maturity, drug pharmacokinetics, and vulnerability to adverse events in early infancy.

While our patient successfully completed the modified desensitization protocol and subsequently demonstrated clinical improvement, causality cannot be definitively established from a single case report. The observed clinical course likely reflects a combination of antimicrobial therapy, supportive care, and the natural progression of pertussis, rather than the desensitization intervention alone. Therefore, this case should be interpreted as an isolated clinical experience rather than evidence supporting broader applicability or generalizable safety and efficacy in this population.

An important consideration is that the patient received diphenhydramine and methylprednisolone following the initial reaction. Although these medications were administered for symptom control, their anti-inflammatory and antihistaminic effects may have partially attenuated subsequent hypersensitivity manifestations during the second desensitization attempt. Therefore, the patient’s apparent tolerance to the modified protocol should be interpreted cautiously, as concomitant pharmacologic suppression of allergic pathways represents a potential confounding factor rather than evidence of complete immunologic tolerance.

## Conclusion

In this specific case of a 51-day-old infant with pertussis who developed an immediate reaction concerning for azithromycin hypersensitivity, and in whom safer age-appropriate alternative antimicrobial options were limited, a modified azithromycin desensitization approach was successfully undertaken in a critical care setting with close multidisciplinary monitoring. This case suggests that, in exceptional and carefully selected circumstances, such an approach may be considered as an individualized management strategy; however, broader conclusions regarding safety, efficacy, or generalizability cannot be drawn from a single case experience.

## CRediT authorship contribution statement

**Almatrafi Mohammed:** Writing – review & editing. **Lein Azzhary:** Writing – review & editing, Data curation. **Loie Goronfolah:** Writing – review & editing, Writing – original draft. **Alohaibi Rayan:** Writing – original draft, Data curation. **Mona Aldabbagh:** Writing – review & editing. **Alghamdi Ahmad:** Writing – review & editing. **Samiyah Althagafi:** Writing – review & editing. **Sara Abed:** Writing – review & editing. **Nouf Alsahaf:** Writing – original draft, Data curation. **Ahmad Almtrafi:** Writing – review & editing, Writing – original draft, Conceptualization. **Bashayer Habib:** Writing – original draft, Data curation.

## Consent

Signed written informed consent was obtained from the patient’s parents and can be submitted upon request.

## Written Informed Consent Statement

Written informed consent was obtained from the patient's parent/legal guardian for publication of this case report and any accompanying images. A copy of the written consent is attached.

## Funding

The authors received no financial support for the research, authorship, and/or publication of this article.

## Declaration of Competing Interest

The authors declare that they have no known competing financial interests or personal relationships that could have appeared to influence the work reported in this paper.

## Data Availability

The data supporting the findings of this case report are available within the article.
